# Immediate and short-term effects of kinesiotaping on muscular activity, mobility, strength and pain after rotator cuff surgery: a crossover clinical trial

**DOI:** 10.1186/s12891-018-2169-5

**Published:** 2018-08-22

**Authors:** Fabienne Reynard, Philippe Vuistiner, Bertrand Léger, Michel Konzelmann

**Affiliations:** 1 Department of Physiotherapy, Clinique romande de réadaptation Suva, Sion, Switzerland; 2 Institute for Research in Rehabilitation, Clinique romande de réadaptation Suva, Sion, Switzerland; 3 Department of Musculoskeletal Rehabilitation, Clinique romande de réadaptation Suva, Sion, Switzerland

**Keywords:** Athletic tape, Electromyography, Rehabilitation, Musculoskeletal disorder, Shoulder

## Abstract

**Background:**

Kinesiotape (KT) is widely used in musculoskeletal rehabilitation as an adjuvant to treatment, but minimal evidence supports its use. The aim of this study is to determine the immediate and short-term effects of shoulder KT on muscular activity, mobility, strength and pain after rotator cuff surgery.

**Methods:**

Thirty-nine subjects who underwent shoulder rotator cuff surgery were tested 6 and 12 weeks post-surgery, without tape, with KT and with a sham tape (ST). KT and ST were applied in a randomized order. For each condition, the muscular activity of the upper trapezius, three parts of the deltoid and the infraspinatus were measured during shoulder flexion, and range of motion (ROM) and pain intensity were assessed. At 12 weeks, the isometric strength at 90° of shoulder flexion, related muscular activity and pain intensity were also measured. Subjects maintained the last tape that was applied for three days and recorded the pain intensity at waking up and during the day.

**Results:**

Modifications in muscle activity were observed with KT and with ST. Major changes in terms of decreased recruitment of the upper trapezius were observed with KT (*P* < 0.001). KT and ST also increased flexion ROM at 6 weeks (*P* = 0.004), but the differences with the no tape condition were insufficient to be clinically important. No other differences between conditions were found.

**Conclusions:**

Shoulder taping has the potential to decrease over-activity of the upper trapezius, but no clinical benefits of KT on ROM, strength or pain were noted in a population of subjects who underwent rotator cuff surgery.

**Trial registration:**

The study was retrospectively registered on ClinicalTrials.gov PRS (NCT03379636) on 21st December 2017.

## Background

Rotator cuff injury is a common source of complaint that leads to pain and decreased function. Symptomatic tears are often repaired surgically. After surgery, physical therapy is necessary to restore shoulder function. Beginning with passive shoulder joint mobility exercises, the treatment becomes more active after 6 weeks, with emphasis on active-assisted to active motion, shoulder proprioception training and sub-maximal isometric exercises. During this phase, resistance work is avoided. From week 13, progressive strengthening is possible, with proprioception and coordination tasks [1].

Rehabilitation protocols often recommend the application of kinesiotape (KT) to decrease pain and enhance motion control. KT is an elastic acrylic adhesive tape, that supports and stabilizes muscles and joints without restricting the range of motion (ROM). The application of KT over manually stretched structures causes the skin to form convolutions that lift the skin. The theory behind this method is that the convolutions facilitate the regeneration of injured tissues by increasing the interstitial space, which allows for increased lymphatic and venous fluid flow. The decrease in pressure between the skin and the underlying connective tissues decompresses subcutaneous nociceptors, leading to decreased pain [2]. Other proposed benefits are 1) on muscular function, by modifying the recruitment activity patterns of the treated muscles and by increasing the strength of weakened muscles; 2) on joint function, by facilitating realignment; and 3) on sensory function, by improving joint position sense and kinaesthetic awareness [2, 3]. Indications for the use of KT are numerous, but scientific evidence remains scarce, with less evidence in favour of KT found with increasing methodological quality of the studies [4–12].

For shoulder pathologies, KT has been used in ten studies between 2007 and 2017 [13–22]. All the studies concerned shoulder impingement syndrome. KT was either compared with sham tape (ST) [13, 16–18, 20, 22], exercises [14, 15], or nonsteroidal anti-inflammatory drugs and sub-acromial corticosteroid injections [19, 21]. Different outcomes were used: questionnaires on upper extremity function and quality of life, pain, ROM and strength. Only one study used electromyography (EMG) to assess muscle activity [13]. The efficacy of KT in shoulder pathologies concern essentially pain [14, 15, 17–19], pain-free ROM [16, 19] and scapular ROM [13]. Improvements were modest in all of these studies and were limited to some spatial and temporal components. To the best of our knowledge, the effects of KT have never been studied after shoulder surgery.

## Methods

### Aim

The aim of this study was to investigate the immediate and short-term effects of KT on shoulder muscle activity, mobility, strength and pain in a population of subjects who underwent rotator cuff surgery. Our hypotheses were that KT would not improve muscle function, mobility, strength or pain in a clinically meaningful way.

### Study design

A controlled crossover study with three treatment arms - no tape (NT) vs KT vs ST - was conducted between January 2013 and October 2016 at the Clinique romande de réadaptation Suva in Sion (Switzerland). A computer block (*n* = 8) randomization process with sealed opaque envelopes was performed to determine the order of passage of the two taping procedures. The physiotherapist who applied the tape was not blinded, but he did not participate in outcome assessment. The main investigator (first author) who collected the data was blinded, as the subjects wore long-sleeved shirts that hid the tape, and the tape was applied behind a folding screen. All subjects provided written informed consent, and all their rights were protected. The study was approved by the regional medical ethics committee (Commission Cantonale Valaisanne d’Ethique Médicale, CCVEM n^o^ 026/12 Sion, Switzerland).

### Subjects

Four local orthopaedic surgeons specializing in shoulder surgery conducted the subject recruitment. The inclusion criteria were as follows: adult subjects who had surgery less than 6 weeks prior after a shoulder rotator cuff tear. The exclusion criteria were as follows: re-tear of the rotator cuff, associated neurological lesion, or concomitant cervical or elbow lesion.

The sample size was calculated a priori using STATA Version 13.1 software (StataCorp, College Station, TX, USA). Based on the data of muscular activity from the study by Hsu et al. [13] and considering a statistical power of 80% and a Type I error of 0.05, a need for at least 36 subjects was necessary to highlight group differences. We enrolled 39 subjects.

### Taping procedure

**Fig. 1:**
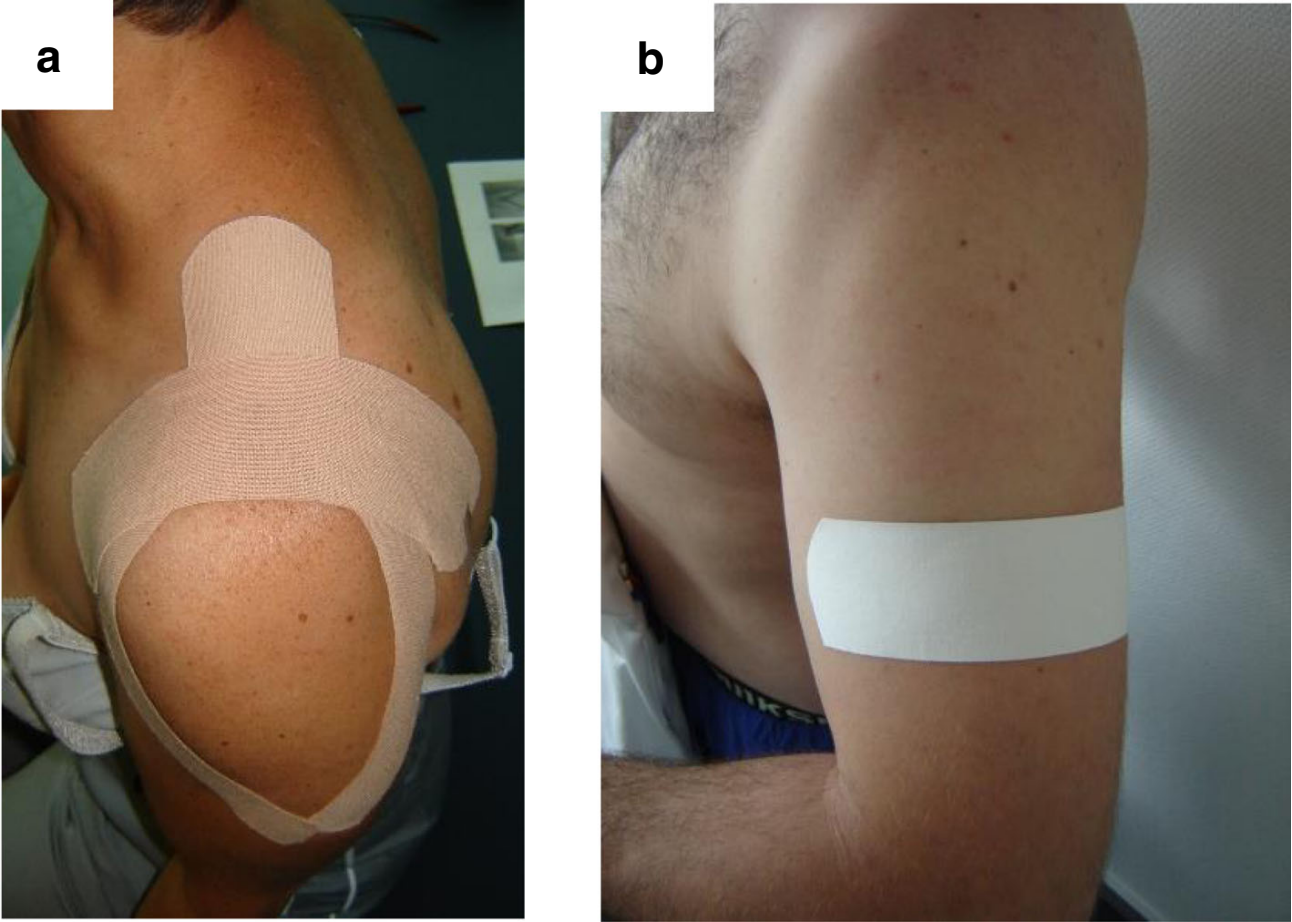
Tape application. **a** Therapeutic kinesiotape application with a Y-strip surrounding the deltoid muscle and an I-strip over the acromioclavicular joint. **b** Sham tape applied under the deltoid tuberosity

A trained physiotherapist applied the two different tapes, a therapeutic KT and an ST, on each subject, as shown in Fig. 1. For KT, elastic beige 5-cm width Leukotape®K (BSN Medical, Hamburg, Germany) was used. It was applied according to the Kase model [2]. The first strip, a Y-strip, was applied with 10 to 15% tension over the deltoid muscle from its origin to insertion, with the first tail along the anterior deltoid while the arm was externally rotated and horizontally abducted. The second tail was applied along the posterior deltoid with the arm horizontally adducted and internally rotated. A second strip, an I-strip, was applied for mechanical correction transversely in the sagittal plane over the acromioclavicular joint with downward pressure applied to the KT, with the arm held along the side.

For the sham condition, rigid Leukotape®Classic (BSN medical, Hamburg, Germany) was used. A 5-cm strip was applied transversely under the deltoid tuberosity with no tension and with no direct influence on shoulder area.

The subjects were informed that two different taping techniques were applied, but they were not given any further details about the taping procedure and effects.

### Testing procedure

**Fig. 2:**
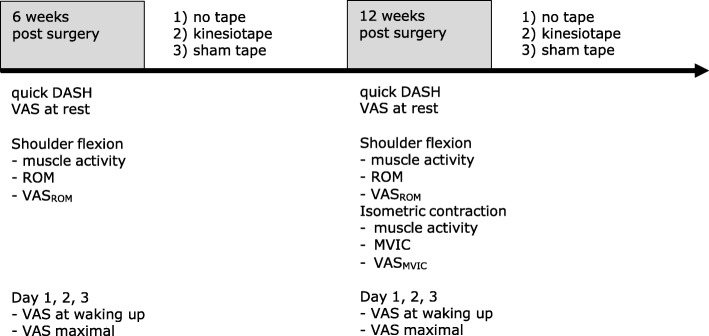
Experimental procedure. Each subject underwent conditions 1), 2) and 3). The sequence of conditions 2) and 3) was randomized. The subjects wore the last tape that was applied for 3 consecutive days

Subjects were assessed on two occasions: 6 and 12 weeks after repair (Fig. 2). Each time, they first answered the French version of the quick Disabilities of the Arm, Shoulder and Hand (DASH) questionnaire in order to assess their physical function and symptoms. This 11-item questionnaire is valid, reliable and responsive in shoulder disorders [23, 24]. They also estimated their pain intensity at rest using a 100-mm visual analogue scale (VAS).

A baseline measurement was then performed without any tape, corresponding to the NT condition. The subjects were seated in a chair without resting on the backrest, with their arms beside their body. Shoulder rotation was neutral, with the elbow extended and the forearm in neutral position. The subjects had to lift their arms in the sagittal plane as high as possible, hold the position for 5 s and then return to the initial position. The movement was repeated once after 1 min of rest.

During the second session at 12 weeks postoperatively, a maximal voluntary isometric contraction (MVIC) measurement was also performed. Measurements were made at 90° of shoulder flexion, neutral rotation, with the elbow extended, the forearm pronated and the fist closed. The strap of the dynamometer was applied at the level of the wrist [25]. The subjects were asked to generate maximal force over a 5-s period. After 1 min of rest, a second trial was performed. The whole session was videotaped for further analysis. At the end of the testing sessions, the subjects were instructed not to remove for 72 h the last tape that was applied.

The sequence always began with the NT condition. Then, the two tapes were applied in a randomized order, and the subjects underwent the same assessment procedures. Group allocation in the KT vs ST group corresponded to the last type of tape the subject had applied, and the tape was worn for three consecutive days.

### Outcome measures

Our primary outcome concerned the activities of important shoulder muscles, i.e., the upper trapezius, the anterior, middle and posterior parts of the deltoid and the infraspinatus. An EMG signal was recorded by a wireless surface EMG system (TeleMyo™ Direct Transmission System; Noraxon, Scottsdale/ US). This system has a baseline noise < 1 μV root mean square, an input impedance > 100 MΩ, a common mode rejection ratio > 100 db and a gain of 400. All EMG signals were recorded at a sampling rate of 1500 Hz with 16 bit-resolution. Prior to electrode application, the skin was prepared according to the SENIAM recommendations [26]. Self-adhesive bipolar silver/silver chloride surface electrodes with 1.75 cm inter-electrode distance (Noraxon Dual Electrodes, Noraxon, Scottsdale/US) were placed in accordance with the SENIAM guidelines [26] and the Criswell [27] proposition (Table 1).

**Table 1:** Electrode placement

Muscles	Electrode placement (location and orientation)
upper trapezius	50% on the line from the acromion to the spine on vertebra C7, in the direction of the line from the acromion to the spine on vertebra C7
anterior part of deltoid	at one finger width distal and anterior to the acromion, in the direction of the line between the acromion and the thumb
middle part of deltoid	line from the acromion to the lateral epicondyle of the elbow, at the greatest bulge of the muscle, in the direction of the line between the acromion and the hand
posterior part of deltoid	two fingerbreadths behind the angle of the acromion, in the direction of the line between the acromion and the little finger
infraspinatus	approximatively 4 cm below the spine of the scapula, over the infraspinatus fossa on the lateral aspect of the muscle, parallel to the spine of the scapula

Muscular activities were recorded and processed with the Myoresearch XP Master Edition 1.08.38 software. For data analysis, EMG signal was bandpass filtered from 10 Hz to 500 Hz using a first order high-pass Butterworth filter at 10 Hz and a low-pass eighth order Butterworth filter at 500 Hz, and the root-mean-square was calculated using a 50-ms moving window. To allow comparison of the EMG signal among different individuals, the data were normalized to a reference voluntary contraction (RVC). This normalization method was used to avoid the appearance of pain that a maximum voluntary contraction would have produced in this group of subjects. The RVC method has demonstrated good reliability [28, 29]. The reference contraction was performed at the beginning of the testing procedure without tape in a seated position with the shoulder abducted at 45° in the scapular plane and the elbow flexed at 90°. The position was maintained for 5 s. After discarding the first and last second and applying the same filtering procedure as described above, normalization processing was performed using a 3-s moving window for the peak of the RVC.

The mean amplitude EMG signal, expressed as a percentage of the RVC, was used to assess muscle activity during isometric conditions at the end range for the mobility test and at 90° for the strength test. As we could not standardize the movement velocity, which is dependent on ROM and pain limitations, the dynamic values of the mobility test, in concentric and eccentric mode, were discarded. As each measure was repeated twice, the mean of both trials was used for analysis. We used a 15% change in muscle activity as the smallest meaningful difference [30].

In terms of secondary outcomes, we investigated active ROM, strength and pain. ROM was measured a posteriori using a video-based motion analysis system (Dartfish 7 ProSuite 7.0 software; Dartfish, Switzerland). The mean of the two trials was recorded. We defined a meaningful clinical change of a 14° difference [31].

Shoulder strength was assessed with MVIC measurements. These measurements were performed with the Isobex 3.0 isometric dynamometer (Medical Device Solutions AG, Oberburg, Switzerland; sampling frequency of 10 Hz, min-max of 0–40 kg, accuracy of 0.1 kg, and measurement threshold of 1.0 kg). The validity of this instrument has been shown in different studies [25, 32, 33]. The mean of the two trials was recorded. To our knowledge, there is no defined minimal clinical change for shoulder flexion isometric strength.

Shoulder pain intensity was assessed with a 100-mm VAS at the end-point of ROM (VAS_ROM_) and after isometric force assessment (VAS_MVIC_). This measurement is known for its excellent reliability [34]. The assessment took place after the two trials of mobility and strength. During the 3 days the subjects had to wear the tape, each day they recorded the pain intensity at waking up (VAS_waking up_) and at the most painful moment during the day (VAS_max_) in a logbook. The activity that was being performed at this time was also noted. For pain intensity, a 20-mm difference was considered a meaningful change [35, 36].

### Statistical analysis

Comparisons between the baseline characteristics of the two groups were computed using nonparametric Mood’s median tests for continuous variables or the chi-squared test for categorical variables.

The outcome variables measured during the mobility and strength tests were compared between the three conditions. For the VAS outcomes during the 3 days after tape application, comparisons were performed between KT and ST alone. As the distributions of the outcome variables were not normal, nonparametric tests comparing the medians (sign tests of matched pairs) were performed. All analyses were conducted using Stata 13.1 software.

As multiple comparisons were performed, the level of statistical significance was set at *P* < 0.017 (0.05/3) for the comparisons between the three tape conditions (comparison of NT vs KT, NT vs ST, and KT vs ST). For comparison between the KT group and the ST group alone, the level of significance was set at *P* < 0.05.

## Results

### Subjects

**Fig. 3:**
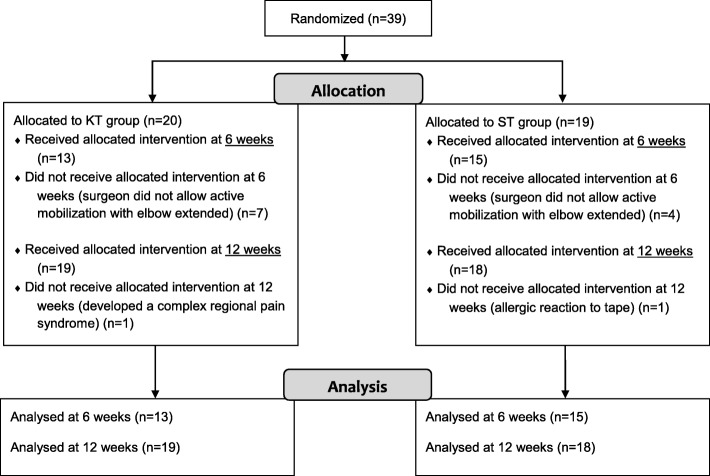
Flow diagram of the progress of subjects throughout the trial

Of the 39 subjects enrolled, 26 underwent both testing sessions. Two subjects were assessed only at 6 weeks, as one subject (group ST) had an allergic reaction to the tape and the other (group KT) thereafter developed a complex regional pain syndrome. Eleven subjects were enrolled only at the 12-week assessment, as their surgeons did not recommend active shoulder mobilization in flexion with the elbow extended at 6 weeks (Fig. 3). The subject characteristics are described in Table 2. There were no significant differences between the groups in terms of gender, age, weight, height, arm tested and the number of tendons repaired (*P* > 0.05). There were also no significant differences in quick DASH and VAS scores at rest between the two groups at 6 and at 12 weeks (*P* > 0.05).

**Table 2:** Subject characteristics

	KT group (*n* = 20)	ST group (*n* = 19)
Subjects assessed (n)		
Only at 6 weeks	1	1
Only at 12 weeks	7	4
At 6 and 12 weeks	12	14
Gender (n)		
Men/women	16/4	14/5
Age, y	59 (9)	60 (10)
Height, m	1.75 (0.08)	1.72 (0.13)
Weight, kg	87.5 (13.5)	78 (35)
Arm tested (n)		
Dominant/non-dominant	12/8	13/6
Number of tendon repair (n)		
1	13	10
2	3	8
3	4	1
Quick DASH		
6 weeks	64 (25)	52 (22)
12 weeks	41 (16)	41 (16)
VAS at rest		
6 weeks	4 (18)	8 (15)
12 weeks	2 (19)	7 (17)

Data are expressed as number (n) or median (interquartile range)

In the 39 surgeries, the supraspinatus was repaired 32 times, the subscapularis 18 times and the infraspinatus 10 times. In 69% of cases, the procedure was completed by an acromioplasty.

The first evaluation was performed an average of 44.2 ± 1.8 days after surgery, and the second was performed 86.0 ± 1.7 days after surgery.

### Muscular activity

**Fig. 4:**
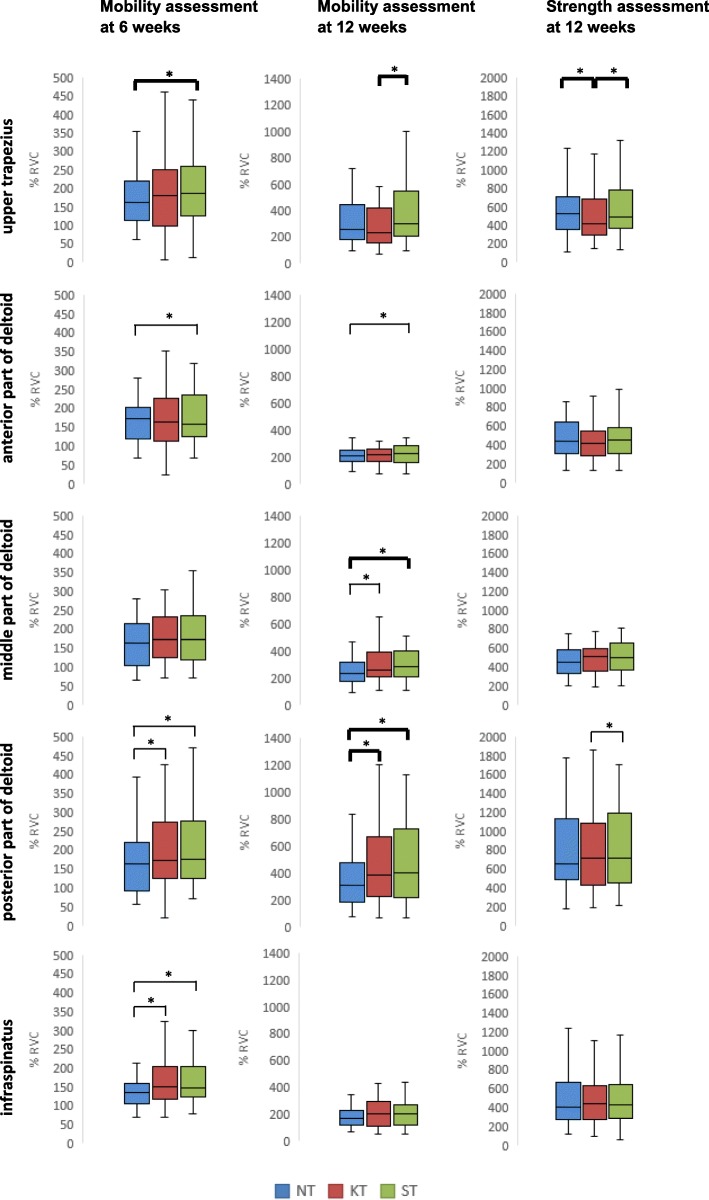
Descriptive statistics of muscle activity. Electromyographic activity of the upper trapezius, the anterior, middle and posterior parts of deltoid and the infraspinatus muscles was recorded. The spread of the data among the subjects is presented with boxplots (median and quartiles). Data are normalized to the reference voluntary contraction (RVC). *significant difference. In bold: statistically and clinically significant difference

Figure 4 summarizes results of the EMG signals. At 6 weeks during active forward flexion, the muscular activity was greater in the KT than in the NT condition for the posterior deltoid (median difference of 7%, *P* = 0.013) and for the infraspinatus (+ 11%, *P* = 0.004). In the ST condition, the muscular activity was also greater than in the NT condition for the posterior deltoid (+ 8%, *P* = 0.001) and the infraspinatus (+ 8%, *P* = 0.001), as well as for the upper trapezius (+ 16%, *P* = 0.013). On the other hand, the activity was lower for the anterior deltoid (− 9%, *P* = 0.004). No difference was found between KT and ST.

At 12 weeks, the muscular activity was greater in the KT than in the NT condition for the middle and posterior deltoid (+ 13% and + 26%, respectively; *P* = 0.001). The ST and NT conditions showed similar results for these two portions of the deltoid (+ 21% and + 33%, respectively; *P* = 0.001). The anterior deltoid also showed increased activation in the ST condition (+ 9%, *P* = 0.004). Comparing the KT and ST conditions, a decreased activity of the upper trapezius was observed with KT (− 31%, *P* = 0.001).

In the strength test, significant differences were found for the upper trapezius, with a decreased activity in the KT condition compared with the NT (− 21%, *P* = 0.016) and the ST (− 19%, *P* = 0.001) conditions. The posterior deltoid showed lower activity in the KT condition than in the ST condition (− 1%, *P* = 0.008), but this difference was negligible.

### ROM

At 6 weeks, active shoulder flexion was statistically greater in the KT condition (P = 0.004) and in the ST condition (P = 0.004) than in the NT condition. However, the magnitude of the median difference was lower, as defined by the minimum clinically important change, with differences of 5.9° and 5.6°. There was no difference between the KT and ST conditions (*P* = 0.04). At 12 weeks, no difference was observed (Table 3).

**Table 3:** Range of motion (ROM), strength (MVIC) and pain (VAS) at 6 and 12 weeks post-surgery

	6 weeks			12 weeks		
	NT	KT	ST	NT	KT	ST
ROM	53.1(36.9)	59.0(40.4)	58.7(35.9)	104.5(68.3)	106.2(47.2)	110.0(53.9)
*P*-value	a) 0.004*	b) 0.004*	c) 0.04	a) 0.90	b) 0.30	c) 0.30
VAS_ROM_	20.5(34.5)	21(30)	23.5(31)	13(20)	9(16)	9(19)
*P*-value	a) 0.20	b) 0.20	c) 0.70	a) 1	b) 0.40	c) 1
MVIC	–	–	–	1.75(1.70)	1.78(1.45)	1.73(1.4)
*P*-value				a) 0.09	b) 0.90	c) 0.20
VAS_MVIC_	–	–	–	16(31)	11(24)	16(31)
P-value				a) 1	b) 0.50	c) 1

Data are expressed as median (interquartile range)

*significant difference

a): NT vs KT; b): NT vs ST; c): KT vs ST.

### Strength

At 12 weeks post-surgery, 7 subjects, 3 in the KT group and 4 in the ST group, could not reach 90° elevation, and the strength test could not be performed. For the remaining subjects, no statistically significant differences were found between the three conditions (Table 3).

### Pain

There were no significant differences in pain values between NT, KT and ST at end-point of active ROM at 6 and at 12 weeks. Pain after MVIC measurements was also similar between conditions (Table 3). Moreover, there were no significant differences either in pain upon awakening or during activity between the KT and ST groups (Table 4). During this follow-up period, activities that triggered the most pain were comparable between the groups and between the two sessions. Physical therapy and home exercise programmes were usually associated with maximal pain, followed by rest, basic activities of daily life and finally more strenuous activities requiring some strength.

**Table 4:** Pain intensity (VAS) during the follow-up period at 6 and 12 weeks post-surgery

		6 weeks			12 weeks		
		KT	ST	*P*-value	KT	ST	*P*-value
VAS_waking up_	Day 1	13 (17)	7 (13)	1	6 (13)	8 (12)	0.70
	Day 2	15 (17)	20 (22)	0.70	5 (15)	9 (15)	0.30
	Day 3	16 (24)	20 (17)	0.70	4 (17)	8 (17)	0.80
VAS_max_	Day 1	28 (22)	26 (24)	0.70	18 (39)	27 (38)	0.70
	Day 2	32.5 (41)	39 (29)	0.80	12 (37)	20 (36)	0.10
	Day 3	27 (35)	34 (38)	0.70	8 (28)	15 (37)	0.20

Data are expressed as median (interquartile range)

## Discussion

In this study, we explored the immediate and short-term effects of shoulder KT on muscular activity, mobility, strength and pain. KT was compared to ST and to NT at 6 and 12 weeks after rotator cuff surgery. These two points in time are key instances in the rehabilitation process, corresponding to the time when rotator cuff loading can be initiated and, at 12 weeks, when initial strengthening can begin. Indeed, at 6 weeks, the remodelling phase has begun, and the collagen network can handle gentle stress; at 12 weeks, the repaired rotator cuff tissue is relatively mature, and tendon-to-bone healing should be able to endure the initiation of strengthening exercises [37].

As expected, we did not find any clinical benefits of KT on ROM, strength or pain. On the other hand, some beneficial effects were observed on muscular activity. Fifteen statistically significant changes among the different muscle groups were observed, but only seven of them were of clinical interest. Furthermore, we failed to demonstrate a clear pattern of changes. Indeed, in the two mobility tests, compared with NT, only the activity of the posterior deltoid was increased at 6 and 12 weeks with KT and with ST, but at 6 weeks the difference was not considered clinically important. As the function of the posterior deltoid is to control flexion by eccentric contraction and to stabilize the shoulder downward and inward [38], this increased activity may improve the coordination of this movement, without having an impact on mobility or pain sensation. Unfortunately, for methodological reasons, we could not analyse the ascending and lowering phases of the flexion movement, which might have given us a better understanding of our disparate results. As we found similar results with KT and with ST for several muscles, we could not determine a beneficial effect of KT. However, it should be noted that compared with ST, the upper trapezius activity at 12 weeks was significantly decreased with KT. The same finding was observed during the strength test, as well as a decreased activity of the upper trapezius compared with NT. This result is consistent with the results of Hsu et al. [13] who also found an inhibition of muscle activity of the upper trapezius with KT. As upper trapezius activity is often increased in shoulder pathology [39–41], its inhibition by KT is of clinical interest. In healthy participants, Lin et al. [42] found also a decreased activity of this muscle with KT as well as a decreased activity of the anterior deltoid, which we did not observe. They stated that the decreased muscular activity was related to proprioceptive feedback and neuromuscular control.

In terms of shoulder mobility, we obtained different findings depending on the period of the session. No difference was noted at 12 weeks, whereas at 6 weeks post-surgery, the KT and ST conditions demonstrated increased flexion amplitude compared with the NT condition. The median difference was less than 6° in both situations, which was not considered clinically important. However, we were surprised to obtain similar effects for KT and ST. This finding suggests that a placebo effect is present and that the slight increase in mobility cannot be considered to be caused by the properties of the KT. Placebo response is known to affect motor performance as well as pain [43]. However, it seems unusual that in our case it affected only ROM and not the pain component. It could also be that the tactile stimulation of the rigid ST, although it was not directly applied to the shoulder area, provided a supportive sensation that facilitated the flexion movement. Among the other studies, only Thelen et al. [16], who compared therapeutic and sham tape application, found an effect of KT on pain-free shoulder mobility in abduction at day one, with an increase of 19°; however, the effect was only transitory. Moreover, no effect of KT was observed for shoulder flexion.

Third, the isometric strength test showed similar performance between conditions, with a maximal median difference of 50 g. The study by Keenan et al. [22] was in accordance with our findings, with no change in isokinetic shoulder rotation strength immediately after KT application. On the other hand, Simsek et al. [18] observed an improvement in shoulder lateral rotation strength 12 days post-tape application, but no difference was observed at 5 days or in other movement directions. In terms of scapular performance, Hsu et al. [13] found no strength differences between KT and ST on the lower trapezius strength. Different studies on knee pathologies have also had mixed results [44–47]. Moreover, the results of the meta-analysis by Csapo and Alegre [48] that evaluated healthy individuals indicated that the potential to increase muscle strength by applying KT was negligible.

Finally, in terms of pain intensity, Lim and Tay [5] proposed various neurophysiological and mechanical mechanisms to explain pain reduction with KT; for example, by the inhibition of the transmission of nociceptive signals, stimulation of the descending pain inhibitory mechanisms, decongestive properties, or by pressure reduction of the subcutaneous nociceptors. However, our results do not allow us to corroborate any of these hypotheses. Indeed, we found no differences among the three conditions at the end range of flexion, during the isometric strength test, or during the follow-up period. Various authors have also studied the immediate or short-term (1 week or less) effects of KT on pain with a population of subjects with shoulder dysfunction [15–18]. Thelen et al. [16] found no effect of KT on pain when assessed immediately or at 3 and 6 days post-application. On the other hand, Shakeri et al. [17] observed decreased pain intensity during movement immediately after KT application. However, the difference between KT and ST was 1.4 points on a 10-point scale, which was not a result that could be considered clinically significant. Moreover, this effect vanished afterwards. Other authors reported decreased pain during activity in the short-term with KT compared with placebo tape [18] or with a physical therapy programme [15]. However, our results do not allow us to recommend the use of KT for the objective of pain reduction.

Our population, which is representative of the usual population undergoing shoulder rotator cuff surgery [49, 50], makes it possible to generalize and apply our results without selection bias. However, this trial has some limitations that need to be addressed. First, at 6 weeks, we did not have the desired number of subjects, as one surgeon thought that it was too early in the rehabilitation process to perform active flexion movements with a large lever arm. Therefore, a larger sample size would be necessary to confirm our results. Another limitation is due to the randomization of the conditions. Indeed, we only randomized the KT and ST conditions, as the protocol always started with NT. This may be the reason why the EMG signals and ROM findings were similar between KT and ST, while they were significantly different from NT. It can be hypothesized that initially the patient mobilizes his arm cautiously and thus gains confidence for the next trial. Third, the normalization procedure for the EMG data was used only in one position for all 5 muscles, so muscle fibre recruitment was unequal between muscles. Moreover, for some subjects, this reference position was effortless to maintain (sub-maximal contraction), whereas it was strenuous (maximal contraction) for others, making the activity level very variable from one person to another. This may influence the results obtained for muscle activity. Finally, we were mainly interested in the immediate effects of KT. Slupik et al. [51] suggested that several hours of tape placement were necessary to obtain a stimulating effect of the KT due to the time necessary for cutaneous mechanoreceptors to improve their neuronal excitability and thus muscle function [52]. It is possible that a longer period of application might be necessary to detect changes. However, we observed no effect on pain intensity at 24, 48 and 72 h of tape application.

## Conclusions

This is the first study to assess KT efficacy on subjects who underwent surgery for shoulder rotator cuff tears. We observed some beneficial effects on muscular activity, particularly a decrease in upper trapezius activity, which is often over-activated, during maximal isometric contraction. The other results are in accordance with literature. We do not have clinical evidence to recommend the use of KT either to increase mobility or muscle strength or to diminish pain during activity. Future studies with a larger sample size are needed to confirm our results, especially those evaluating the effect of KT on muscular recruitment.

## Abbreviations

DASH: Disabilities of the arm shoulder and hand
EMG: Electromyogram
KT: Kinesiotape
MVIC: Maximal voluntary isometric contraction
NT: No tape
ROM: Range of motion
RVC: Reference voluntary contraction
ST: Sham tape
VAS: Visual analogue scale for pain intensity
VAS_max_: Maximal pain intensity
VAS_MVIC_: Pain intensity after isometric force assessment
VAS_ROM_: Pain intensity at end point of range of motion
VAS_waking up_: Pain intensity at waking up
